# *Listeria monocytogenes* is a solvent tolerant organism secreting a solvent stable lipase: potential biotechnological applications

**DOI:** 10.1007/s10529-022-03284-5

**Published:** 2022-08-25

**Authors:** Priyanka Priyanka, Gemma K. Kinsella, Gary T. Henehan, Barry J. Ryan

**Affiliations:** School of Food Science and Environmental Health, Technological University Dublin, Grangegorman, Dublin 7, D07 ADY7 Ireland

**Keywords:** Extracellular lipase, *Listeria monocytogenes*, Polyester degradation, Solvent tolerant, Wastewater

## Abstract

**Purpose:**

The emerging biobased economy will require robust, adaptable, organisms for the production and processing of biomaterials as well as for bioremediation. Recently, the search for solvent tolerant organisms and solvent tolerant enzymes has intensified. Resilient organisms secreting solvent stable lipases are of particular interest for biotechnological applications.

**Methods:**

Screening of soil samples for lipase-producing organisms was carried out on Rhodamine B plates. The most productive lipase-producing organisms were further screened for their resistance to solvents commonly used in biotechnological applications.

**Results:**

In the course of screening, one of the isolated organisms that exhibited extracellular lipase activity, was identified as the human pathogen *Listeria monocytogenes* through 16S rRNA sequencing. Further exploration revealed that this organism was resistant to solvents ranging from log P − 0.81 to 4.0. Moreover, in the presence of these solvents, *L. monocytogenes* secreted an extracellular, solvent tolerant, lipase activity. This lipase retained approximately 80% activity when incubated in 30% (v/v) methanol for 24 h.

**Conclusion:**

These findings identify *L. monocytogenes* as a potentially useful organism for biotechnological applications. However, the fact that *Listeria* is a pathogen is problematic and it will require the use of non-pathogenic or attenuated *Listeria* strains for practical applications. Nonetheless, the ability to adapt to rapidly changing environmental conditions, to grow at low temperatures, to resist solvents and to secrete an extracellular solvent tolerant lipase are unique and highly useful characteristics. The potential application of *L. monocytogenes* in wastewater bioremediation and plastics degradation is discussed.

**Supplementary Information:**

The online version contains supplementary material available at 10.1007/s10529-022-03284-5.

## Introduction

The shift away from a petroleum-based raw materials economy to a biobased economy will require systems innovations. It is likely that robust, solvent tolerant, organisms will be central components of emerging technologies for the sustainable production and processing of biomaterials (Mohedano et al. 2022; Schalck et al. 2021; Kusumawardhani et al. 2018). Currently, significant effort is being expended to either identify new solvent tolerant organisms or to engineer existing organisms for specific solvent environments (Mohedano et al. 2022; Tian et al. 2022; Kyoungseon et al. 2021; Schalck et al. 2021; Srivastava et al. 2021; Wang et al. 2021; de Carvalho et al. 2019; Wynands et al. 2019). Extensive engineering of *Pseudomonads*, for example, has yielded a solvent stable *iso-*butanol (biofuel) producing strain and a strain capable of converting cyclohexane to 6-hydroxyhexanoic acid, a polycaprolactone monomer (Ankenbauer et al. 2021; Bretschneider et al. 2021). Another area that requires robust solvent tolerant organisms is the processing of wastewater; specifically, there is a need for organisms that produce extracellular lipases to degrade wastewater lipids (Aktar et al. 2021; Priyanka et al. 2019a, 2019b).

Concurrently, there is significant interest in the application of solvent stable lipases, and closely-related cutinases, in a variety of areas (Ismail et al. 2021; Priyanka et al. 2019a, 2019b) including; plastics breakdown in the environment, ester synthesis and biodiesel production (Singh et al. 2019; Imanparast et al. 2018; Malekabadi et al. 2018). Lipases have also been applied to the treatment of lipid-rich wastewaters to alleviate environmental problems associated with high lipid loads (Nimkande and Bafana 2022; Patel et al. 2020; Boran et al. 2019). Thus, fats, oil and grease (FOG), as triglycerides, are discharged in wastewater from food industries, oil refineries, meat processing plants, cosmetics and pharmaceuticals industries. Dairy wastewater, for example, contains 8,288 mg/L of FOG. The FOG content of oil refineries, food packaging and domestic wastewater ranges between 110–264,150; 100–1,000 and 50–100 mg/L respectively. Untreated lipids from these industries form an oily layer on water preventing oxygen and sunlight penetration thereby harming aquatic ecosystems. These lipids can also accumulate to block sewer lines and hinder treatment processes (see Nimkande and Bafana (2022) for review).

Identifying sources of appropriate lipases is an ongoing area of research and even pathogenic bacteria such as *Listeria monocytogenes* are now being re-examined for their application potential supported by enhanced sequence and metagenomic approaches. *Listeria monocytogenes* is a Gram-positive foodborne pathogen that causes listeriosis in humans. It is an unusually robust and highly adaptable organism with the ability to grow over a wide range of temperatures, from 4 °C to 45 °C, and to thrive in a wide variety of ecological niches (Lopes-Luz et al. 2021, Ingeborg et al. 2011). The ability to adapt to changing temperatures is largely due to its capacity to rapidly alter membrane composition in response to stressors (Najjar et al. 2007). The response to stress is mainly controlled by the alternative sigma factor SigB (σB), which influences environmental survival within the gastrointestinal tract and virulence (Dorey et al. 2019; Guerreiro et al. 2020). *L. monocytogenes* is known to secrete lipolytic enzymes to break down host cell membranes during cell invasion (Smith et al. 1995).

Ongoing studies in this laboratory have sought to identify solvent tolerant enzymes for biotechnological applications (Priyanka et al. 2019a, 2019b, 2020). While screening for solvent tolerant lipase-producing organisms from soil samples it was observed that *Listeria monocytogenes,* enriched from soil and confirmed by 16S rRNA sequencing, was stable in the presence of common solvents. In this report we suggest that it is the unique robustness of *Listeria* that allows it to grow over a wide range of temperature and pH values, combined with its solvent tolerance, that makes it of interest for biotechnological applications.

## Materials and methods

### Chemicals and materials

All chemicals were analytical grade and were purchased from Sigma-Aldrich.

### Enzyme assays

#### Plate assay

Rhodamine B agar plates were used for the detection of lipolytic activity from microbial strains. Rhodamine B agar plates (20 ml volume) were prepared using the method described by Kouker and Jaeger 1987).

#### Spectrophotometric assay

*p*-NPP (*para-*-Nitrophenol Palmitate) was used as the substrate for the estimation of lipase activity as per Glogauer et al., (2011). Lipase activity was measured after 30 mins of incubation at 28°C. Liberated *para-*-Nitrophenol was monitored by its absorbance at 410nm. Briefly, a 20 mM stock solution of p-NPP (Stock A) was prepared in a 1:4 ratio of Acetonitrile:Isopropanol. Stock B, containing Tris-HCl, CaCl_2_ and Triton X-100 at pH 7.5, was used to prepare the substrate for the lipase assay. The substrate was prepared directly before the assay by the addition of Stock A to a preheated (60 °C) Stock B, under continuous stirring. Then, 0.54 mL of Stock A was added to 9.46 mL of stock B to achieve a final concentration of components as follows: 50 mM Tris-HCl, pH 7.5, 1 mM CaCl_2_, 0.30% (v/v) Triton X-100, 1 mM p-NPP. To initiate the reaction, 230 µL of assay substrate was added to 20 µL of lipase sample, in triplicate, in a sterile flat bottom U-shaped 96-well plate. The mixture was incubated at 28 °C for 30 min. After incubation, the absorbance was measured at 410 nm using a Powerwave Microplate spectrophotometer.

### Isolation and identification of solvent tolerant lipase producing strains

#### Sample enrichment and culture

Soil samples, collected from various locations in the Wicklow mountains in Ireland, were cultured in enrichment media (Priyanka et al. 2019) for 72 h at 28 °C, 200 rpm. The supernatants of the enriched samples were serially diluted (10^−1^ to 10^−11^) with autoclaved double distilled water. 100 μl of each diluted sample was spread on Rhodamine B plates and incubated at 28°C for 48 h. Individual lipase producing colonies were aseptically picked from the Rhodamine B plates and were sub-cultured 10 times on LB agar plates at 28 °C until pure colonies were isolated. The pure colonies were grown in LB media overnight at 28 °C, 200 rpm and were re-screened for the presence of lipase by streaking on Rhodamine B plates and statically incubating the plates at 28 °C overnight (16 h). The stability of lipase producing cultures in different solvents was determined by a “Plate overlay method” (see 2.3.2) using various solvents ranging from log P < 0.2 to log P > 2.

#### Plate overlay method

The method described by Patel et al., (2014) was used. Briefly, pure cultures of lipase producing strains were grown overnight (16 h) at 28 °C, 200 rpm. 20µL of this culture was spot inoculated onto an LB agar plate and allowed to dry in a sterile laminar flow hood for 30 min. The plates were then overlaid with 10 ml organic solvent and incubated at 28 °C for 6 h. Excess solvents were then removed with sterile tips and the plates incubated overnight at 28 °C to observe any growth in the spotted cultures. The ability of the cultures to grow and produce lipase following solvent exposure was observed.

#### Isolate identification

Lipolytic strains stable in multiple solvents were 16S rRNA sequenced (Eurofins, Germany).

### Lipase production

#### Fermentation methods

1–15% (v/v) of an overnight grown culture in LB media (pH 7.5) was added to basal lipase production media containing 50 g/L bacteriological peptone, 2 g/L sodium chloride, 0.4 g/L magnesium sulfate, 0.5 g/L ammonium sulfate, 0.3 g/L dipotassium hydrogen phosphate, 0.03 g/L potassium hydrogen phosphate and 10 g/L olive oil at pH 7.0 ± 0.2. After every 24 h of fermentation, the cell free supernatant was analysed for lipolytic activity by the spectrophotometric assay (see“Spectrophotometric assay”).

## Results

### Biodiscovery of lipase-producing solvent tolerant organisms

**Fig. 1:**
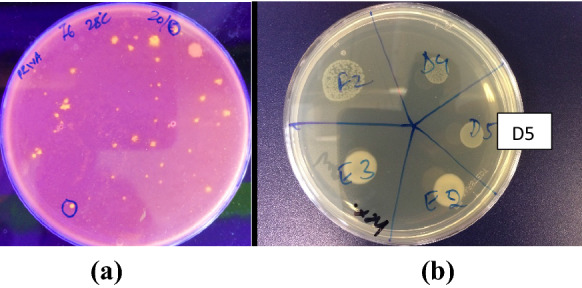
Screen for solvent tolerant organisms secreting extracellular lipase activity: **a** Example of initial screening of soil samples for lipase producing organisms. UV illuminated olive oil-Rhodamine B agar plate with serially diluted (10^–6^) enriched soil media. Orange-pink fluorescence emitting colonies that confirm the production of lipase activity by these strains appeared after 2 days of incubation at 28 °C. Plates were prepared as described in Methods (see “Plate assay”). **b** Example of growth of strains in the presence of solvent (*n*-hexane in this case) using a plate overlay method (see “Plate overlay method” for the method). The organism annotated as D5 was subsequently identified as *L. monocytogenes*. The other organisms shown on this plate were not further characterized

Soil samples were screened for extracellular lipase production in a medium containing triglyceride and Rhodamine B (see Supplemental Table 1 for a list of sampling sites and coding of samples). This method relies on hydrolysis of lipids to release fatty acids which react with the Rhodamine B dye to form fluorescent complexes. Figure 1a shows an example of initial screening of a soil sample on a Rhodamine B plate with lipase-producing colonies forming fluorescent halos around the colonies. The size of the fluorescent halo is proportional to lipase activity. Figure 1b shows an example of the growth of some of these organisms in the presence of neat *n*-hexane.

At the outset, 36 organisms that displayed extracellular lipase activity were identified on Rhodamine B plates. Single colonies were prepared and cultured to an OD of 0.6 and a 20 µl sample of each was inoculated onto a Rhodamine B plate. Of these, 25 showed evidence of sufficient lipase production (a fluorescent halo of greater than 1 cm diameter was arbitrarily chosen as a cut-off point) to justify further exploration (see Supplemental Table 2). All 25 were examined for growth in the presence of solvents (see Table 1). The log P is a measure of the hydrophobicity of a solvent. A solvent with a low log P will have greater water solubility. Log P determines a solvent’s toxicity towards a microbe and solvents with lower log P will generally have a greater detrimental effect (Segura et al. 2012). These are solvents such as ethanol, methanol and isopropanol. Thus, these were used at levels of 20% (v/v) in this study since higher levels significantly inhibited growth. Table 1 shows that 8 cultures (A1, A2, I3 A3, D1, D5, H1 and H3) were stable in a broad range of solvents. In a preliminary test, the cultures A1, A2 and I3 showed the lowest activity in 30% (v/v) methanol (data not shown) and were not explored further.

**Table 1:** Test for growth of lipase producing organisms in selected solvents categorized by hydrophobicity (log P)

Solvent nature	Solvent	Log P	% (v/v)	Organisms showing growth in solvent
Non-polar/ Hydrophobic	Ethyl acetate	0.68	100	–
	Toluene	2.5	100	–
	Cyclohexane	3.2	100	A1, A2, A3, D1, D5, H1, H3, I3
	*n*-Hexane	3.5	100	A1, A2, A3, C3, D1, D2, D3, D4, D5, E2, E3, F2, H1, H3, I3
	Heptane	4.0	100	A1, A2, A3, C3, D1, D2, D3, D4, D5, E2, E3, F2, H1, H3, I3
	Isopropanol	0.54	50	–
Polar/ hydrophilic	Ethanol	− 0.18	20	A1, A2, A3, A4, B1, B2, C3, D1, D2, D3, D4, D5, F2, F3, G1, H1, H3, I3
	Methanol	− 0.81	20	A1, A2, A3, A4, B1, B2, C3, D1, D2, D3, D4, D5, F2, F3, G1, H1, H3, I3

The coding refers to the sites from which the organisms were sourced (see Supplemental Table 1)

Five cultures (A3, D1, D5, H1 and H3) showed growth in the broadest range of solvents were selected for further characterisation. In this study, an incubation time of 6 h in the respective solvent was considered sufficient to challenge the microbial colonies. This aligned with previous research where 6 h of solvent treatment was sufficient to identify solvent stability in *Pseudomonas aeruginosa* in the presence of *n*-hexane, *n*-heptane, styrene, xylene isomers and ethylbenzene (Lazaroaie, 2009).

### 16S rRNA sequencing of cultures

Five solvent stable, lipase-producing cultures (A3, D1, D5, H1 and H3) which demonstrated growth in the presence of a number of solvents were examined by 16S rRNA sequencing using a commercial service (Eurofins, Germany). The sequences (see Supplemental data) were compared to those in NCBI database by nBLAST. The sequencing data revealed A3 as a Pseudomonas sp. BIM B-86, D1 as a Sphingomonas sp., D5 as *Listeria monocytogenes*, H1 as *Pseudomonas reinekei* and H3 as *Pseudomonas brenneri.* Studies of the solvent stable enzymes from *P. brenneri* and *P. reinekei* have previously been reported (Priyanka et al. 2019a, b, 2020). The finding that *L. monocytogenes* was among the solvent tolerant organisms identified by the screening process was somewhat surprising since no reference to this property of *Listeria* was found in the literature.

### Solvent stable lipase activity

**Fig. 2:**
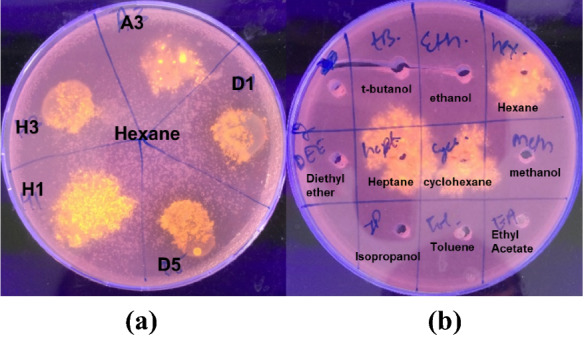
Solvent stability of secreted extracellular lipase activity. UV-illuminated Rhodamine B agar plates **a** cell free supernatant of cultures (A3, D1, D5, H1 and H3) treated with *n*-hexane by plate overlay method; **b** typical screen of cell free supernatants of a H3 culture treated with 50% (v/v) of various organic solvents for 24h at 28°C. The presence of fluorescence in **a** and **b** indicates the stability of crude lipases towards *n*-hexane, *n*-heptane and cyclohexane. The coding for these samples is shown in Supplemental Table 1

The five promising isolates that were identified were further examined for extracellular lipase solvent stability. The cell free supernatants of these five, containing the secreted lipase activity, were tested against a range of solvents ranging from log P < 0.2 to log P > 2 by the plate overlay method (see Table 1). Figure 2a shows that the extracellular lipase activities produced by these strains are *n*-hexane tolerant. Figure 2b shows a typical screening for solvent resistant lipase activity using Rhodamine B plates.

**Fig. 3:**
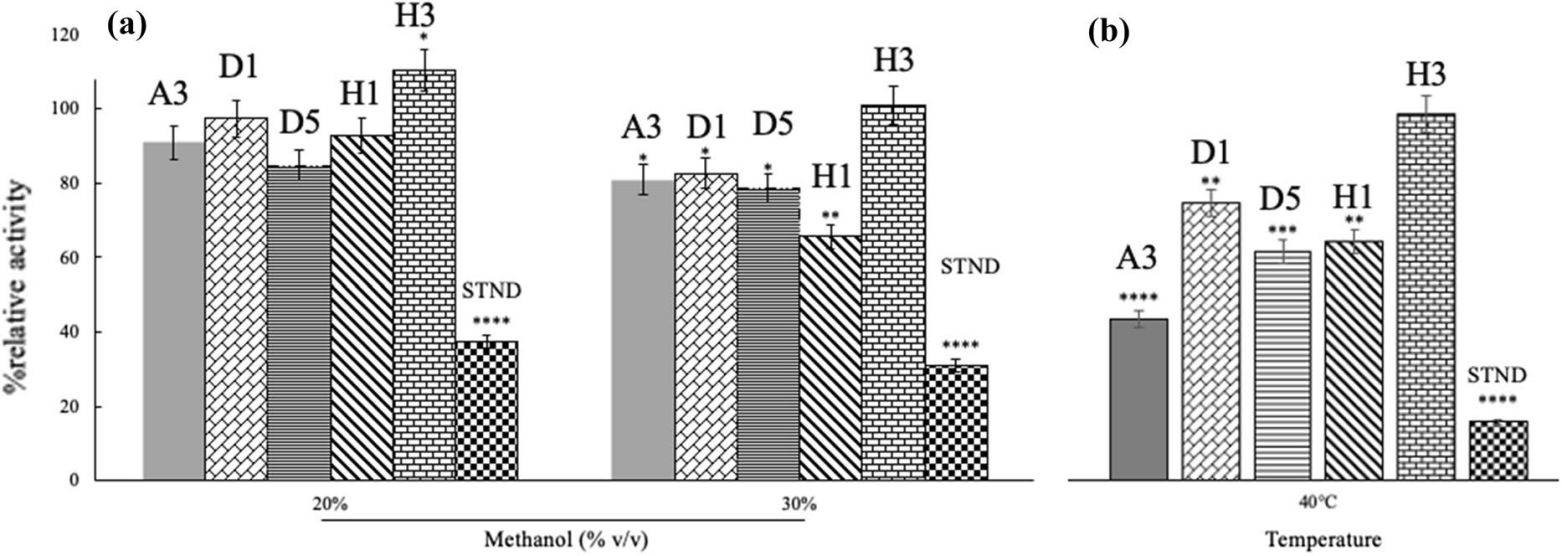
The relative activity of extracellular lipase activity when treated with 20% (v/v) and 30% (v/v) methanol for 24 h at 28 °C and 40 °C. **a** Shows relative activity after incubation at 28 °C in 20% (v/v) and 30% (v/v) methanol while (**b**) shows 30% (v/v) methanol at 40 °C for 24 h. All activities were expressed as a percentage of the activity in the absence of methanol. A sample of 30 mg/mL of porcine pancreas lipase (Aldrich) was used as a standard for comparative purposes (denoted STND in the graph). The assay was performed in triplicate using *p*-NPP as substrate and relative activity was calculated by comparing the activity of lipase in 20% (v/v) and 30% (v/v) methanol at different temperatures to a sample with no methanol at 28 °C or 40 °C. Data represented here are the mean of three independent experiments with error bars indicating standard deviation. (****, ***, **, * represents significance changes in activity where ****p < 0.0001; ***p = 0.0001–0.001; **p = 0.001–0.01, *p = 0.01–0.05 by t-test)

In addition to the plate overlay data, it was necessary to directly quantify the effects of each solvent on enzyme activity. Figure 3a shows treatment of cell free supernatants with methanol. In this experiment the crude lipase extracts were treated with either 20% (v/v) or 30% (v/v) methanol for 24 h at 28 °C. Methanol was chosen since it is one of the solvents that showed greatest ability to inhibit cell growth.

It was clear that, while not quite as robust as the other strains tested, the *L. monocytogenes* (D5) extracellular lipase showed good stability in the presence of methanol retaining approximately 80% of its activity after incubation in 30% (v/v) methanol for 24 h. A lipase standard, the lipase from porcine pancreas, was used as a comparator and showed much greater sensitivity to methanol than the lipases identified by the screening methods employed. Additionally, the *L. monocytogenes* enzyme displayed good resistance to an increase in temperature (see Fig. 3b). 

## Discussion

In these studies, our objective was to identify solvent tolerant organisms secreting solvent stable lipases. The initial screen of soil samples identified a number of interesting isolates, among them the human pathogen *L. monocytogenes*. The fact that this organism was solvent tolerant has not been reported previously and is, potentially, a highly important characteristic. Solvent tolerant organisms are useful for production of biomaterials (Mohedano et al. 2022; Kusumawardhani et al. 2018). The ability to survive solvent exposure can be especially useful for two-phase fermentation systems (Heipieper et al. 2007) and biofuels production (Nicolaou et al. 2010). Moreover, such solvent tolerance has a range of potential applications in bioremediation (e.g. Gao et al. 2020).

Somewhat surprisingly, *Listeria* was not tolerant to toluene (Log P = 2.5). Some *Pseudomonads*, for example, show very high resistance to toluene being able to tolerate saturating levels (Molina‐Santiago et al. 2017). *Listeria* also showed no tolerance to the ester ethyl acetate: it is possible that the excreted lipase (which is essentially an esterase) is inhibited by ethyl acetate and that this inhibition served to curtail *Listeria* growth. This toluene and ethyl acetate sensitivity indicates that *Listeria* may not be suitable for some biobased applications, such as biofuels production. In common with many bacteria, *L. monocytogenes* was also not stable in greater than 30% (v/v) methanol or similar solvents with a low log P. Despite these limitations, this organism could be useful for applications where a robust extracellular lipase was required, such as in the breakdown of environmental polyester plastics and lipid breakdown in wastewater streams (Aktar et al. 2021; Tan et al. 2021).

Examination of the cell free supernatant of *L. monocytogenes* cultures showed that its extracellular lipase activity was also quite stable to solvent exposure and a significant increase in temperature did not reduce its solvent tolerance. This finding has not been previously reported and may be an important factor in the survival of the organism. This lipase activity is, itself, of potential biotechnological interest and could be cloned and directly used in applications such as polyester degradation, environmental lipid breakdown or biocatalysis (Priyanka et al. 2019a, 2019b).

It is of some interest that the growth of *Listeria* is well known to be inhibited by certain esters, such as monoacylglycerols, quercetin esters and 4-hydroxyphenylacetic acid, all of which are potentially lipase inhibiting compounds (Wang et al. 1993; Gatto et al. 2002; Shi et al. 2021). It is tempting, therefore, to suggest that its extracellular lipolytic activity may be growth regulating. Thus, the extracellular lipase of this organism may be useful as a target to inhibit *Listeria* growth.

While solvent tolerance of *Listeria* has not, to the best of our knowledge, been specifically described previously, its robustness and ability to withstand cleaning agents is known, therefore, this solvent tolerance is not entirely surprising (Wiktorczyk-Kapischke et al. 2021). However, the fact that its extracellular lipase is solvent stable has not been previously reported.

The exact identity of the lipase(s) secreted in the presence of solvents is not known with certainty although the secretion of lipases by this organism is well-known (Mohedano et al. 2022; Kusumawardhani et al. 2018). Two extracellular lipase activities have been described for *L*. *monocytogenes* and one of these is a broad range phospholipase C (Smith et al. 1995) that is essential for cell to cell spread of the pathogen. 

Finally, the fact that *Listeria* can withstand a solvent treatment step during its isolation from soils may be useful. Thus, in environments where *Listeria* isolation is challenging due to its presence at low amounts among other bacteria, it may be helpful to be able to use a solvent step to selectively enrich for *Listeria* species.

While further studies are clearly needed, this work indicates that this organism may potentially find application in wastewater treatment, where lipid accumulation causes severe problems for waste degradation (Aktar et al. 2021). The ability of *Listeria* to grow at low temperatures and low pH in the presence of high amounts of salt is particularly advantageous in this regard. Another potential area of application is in the area of plastic degradation in soils where low temperature growth is a significant hindrance to other organisms (Zhang et al. 2022). However, since the cultivation of a pathogen for biotechnological purposes is problematic, it will be necessary to explore the use of non-pathogenic strains (Mohedano et al. 2022) or to alter strain pathogenicity by ablation of virulence genes (Shi et al. 2021).

For many organisms, the requirement to operate in real world conditions where temperature control is not available is a significant challenge. The adaptability of *Listeria* would appear to offer significant advantages for such application areas. There are looming environmental problems in the treatment of wastewater with high lipid loads and in environmental polyester degradation (even for so-called biodegradable plastics). The adaptability of Listeria would appear to offer significant advantages for such application areas. The findings reported herein may stimulate further exploration of this organism for sustainable, biotechnological applications.

## Supplementary Information

Below is the link to the electronic supplementary material.

Supplementary file1 (DOCX 30 kb)

Supplementary file2 (DOCX 13 kb)

Supplementary file3 (DOCX 12 kb)
