# Supplementary material for: Listeria monocytogenes is a solvent tolerant organism secreting a solvent stable lipase: potential biotechnological applications
Source: Biotechnol Lett. 2022 Aug 25;44(10):1139–47. doi: 10.1007/s10529-022-03284-5 (PMC9481501; doi:10.1007/s10529-022-03284-5)
Supplement: Supplementary file 1 — Supplementary file1 (DOCX 30 kb) [file 10529_2022_3284_MOESM1_ESM.docx]

Supplemental Material

Table S.1: Location of soil sample sites and annotation of lipase-producing colonies picked from the Rhodamine B agar plates after 2 days of incubation at 28°C. These soil samples were gathered from sites using sterile methods and transported to laboratories in Technological University Dublin for screening on Rhodamine B plates. Colonies were named based on the source of the soil sample. (Co. is county).

| **Soil sample** | **Source of Isolation** | **Selected lipase producing colonies** | **Nomenclature** |
| --- | --- | --- | --- |
| A | Co. Laois (Garage) | 5 | A1, A2, A3, A4, A5 |
| B | Co. Laois (Garage) | 4 | B1, B2, B3, B4 |
| C | Co. Laois (Garage) | 5 | C1, C2, C3, C4, C5 |
| D | Wicklow Mountains | 5 | D1, D2, D3, D4, D5 |
| E | Wicklow Mountains | 3 | E1, E2, E3 |
| F | Wicklow Mountains | 3 | F1, F2, F3 |
| G | Wicklow Mountains | 3 | G1, G2, G3 |
| H | Wicklow Mountains | 3 | H1, H2, H3 |
| I | Dublin City (Garage) | 5 | I1, I2, I3, I4, I5 |
