# Supplementary material for: Listeria monocytogenes is a solvent tolerant organism secreting a solvent stable lipase: potential biotechnological applications
Source: Biotechnol Lett. 2022 Aug 25;44(10):1139–47. doi: 10.1007/s10529-022-03284-5 (PMC9481501; doi:10.1007/s10529-022-03284-5)
Supplement: Supplementary file 2 — Supplementary file2 (DOCX 13 kb) [file 10529_2022_3284_MOESM2_ESM.docx]

Supplemental material

Table S.2: Lipase producing colonies which displayed a >1cm diameter of fluorescence halo on Rhodamine B agar plate after 16h of incubation at 28°C. Supplemental Table 1 shows the sites at which these samples were collected.

| **Soil sample** | **Number of Lipase producing strains isolated** | **Significant lipase producing strains** | **Strains selected** |
| --- | --- | --- | --- |
| A | 5 | 4 | A1, A2, A3, A4 |
| B | 4 | 2 | B1, B2 |
| C | 5 | 3 | C1, C2, C3 |
| D | 5 | 5 | D1, D2, D3, D4, D5 |
| E | 3 | 2 | E2, E3 |
| F | 3 | 2 | F2, F3 |
| G | 3 | 1 | G1 |
| H | 3 | 2 | H1, H3 |
| I | 5 | 4 | I2, I3, I4, I5 |
