# Supplementary material for: Listeria monocytogenes is a solvent tolerant organism secreting a solvent stable lipase: potential biotechnological applications
Source: Biotechnol Lett. 2022 Aug 25;44(10):1139–47. doi: 10.1007/s10529-022-03284-5 (PMC9481501; doi:10.1007/s10529-022-03284-5)
Supplement: Supplementary file 3 — Supplementary file3 (DOCX 12 kb) [file 10529_2022_3284_MOESM3_ESM.docx]

**Supplemental Data: 16S rRNA sequences obtained from commercial sequencing service Eurofins (Germany): 27F (forward primer)**

Five cultures isolated from soils showed solvent tolerance in a wide range of solvents. These were the isolates labelled as A3, D1, D5, H1, H3 (see Supplemental Table 1 for sample annotation).

The 16S rRNA sequences of these cultures were obtained from a commercial sequencing service and were used to identify the solvent tolerant organisms isolated from soil samples. The sequences were compared to those in NCBI database by nBLAST. The sequencing data revealed A3 as a *Pseudomonas sp.* BIM B-86, D1 as a *Sphingomonas* sp., D5 as *Listeria monocytogenes*, H1 as *Pseudomonas reinekei* and H3 as *Pseudomonas brenneri* .

**Figure S.3:** 16S rRNA sequencing obtained from Eurofins Germany revealed 99% homology of A3 with 16S rRNA of *Pseudomonas* sp. BIM B-86; 84% homology of D1 with *Sphingomonas* sp. PP-2; 99% homology of D5 with *Listeria monocytogenes* strain J1926; 99% homology of H1 with *P.reinekei* SN8; 98% homology of H3 with *P.brenneri*.

**>Culture_A3_**

GGTGGCGGCAGCTACACATGCAGTCGAGCGGATGAGAGGAGCTTGCTCCTGGATTCAGCGGCGGACGGGTGAGTAATGCCTAGGAATCTGCCTGGTAGTGGGGGACAACGTTTCGAAAGGAACGCTAATACCGCATACGTCCTACGGGAGAAAGCAGGGGACCTTCGGGCCTTGCGCTATCAGATGAGCCTAGGTCGGATTAGCTAGTTGGTGAGGTAATGGCTCACCAAGGCGACGATCCGTAACTGGTCTGAGAGGATGATCAGTCACACTGGAACTGAGACACGGTCCAGACTCCTACGGGAGGCAGCAGTGGGGAATATTGGACAATGGGCGAAAGCCTGATCCAGCCATGCCGCGTGTGTGAAGAAGGTCTTCGGATTGTAAAGCACTTTAAGTTGGGAGGAAGGGCAGTAACTTAATACGTTGCTGTTTTGACGTTACCGACAGAATAAGCACCGGCTAACTCTGTGCCAGCAGCCGCGGTAATACAGAGGGTGCAAGCGTTAATCGGAATTACTGGGCGTAAAGCGCGCGTAGGTGGTTCGTTAAGTTGGATG

TGAAATCCCCGGGCTCAACCTGGGAACTGCATTCAAAACTGTCGAGCTAGAGTATGGTAGAGGGTGGTGGAATTTCCTGTGTAGCGGTGAAATGCGTAGATATAGGAAGGAACACCAGTGGCGAAGGCGACCACCTGGACTGATACTGACACTGAGGTGCGAAAGCGTGGGGAGCAAACAGGATTAGATACCCTGGTAGTCCACGCCGTAAACGATGTCAACTAGCCGTTGGGAGCCTTGAGCTCTTAGTGGCGCAGCTAACGCATTAAGTTGACCGCCTGGGGAGTACGGCCGCAAGGTTAAAACTCAAATGAATTGACGGGGGCCCGCACAAGCGGTGGAGCATGTGGTTTATTCGAGCAACGCGAGAACCTTACCAGGCCTTGACATCCAATGACTTTCAGAGATGATGTGCCTCGGGAACATTGAGACAGTGCTGCATGGCTGTCGTCAGCTCGGTGTCGTGAGATG

**>Culture_D1_**

CTGCGCTGCTACCATGCAGTCGAGCGGCAGCATCGGCCTTTGTGCCGCATGGTGCGTACGCATGTGAAACTGCCCTCATGTTTCCAACTCACCGAGGGATTGCTGCTCGACAGGATGATATCCCCAAATCACATTTTATCGCATGAAGATAAGCATCTTCAGACCTCACTCTCATAGTGTGATCTAGCCGCTTTTAGCGACAATCCTTAGCTGGTCTGACAGGAGAAGCTCCCCACTGGGACTGAGACGGAAGCCCCACTCCTCTGGGAGGGACCCATGGGGAATATTGAACAATGGGCGAACACCTGATCCTATTGGCGGCGTGAGTGTTAAAAGCCTTAGGGTTGTAAAGCTCTTTTACCCGGGATGATAATGACAATACCGGGATAAAAAACTCCGGCTATCTCCTTGCCCCCACCCCCGGTAATACTGAAGGAGCTATCGTTATTCGCAATTACTGTGCGCAAAGCGCACGTAGGCGGCTTTGTGTGTTAGAGGTGAAAACTCGGAGCTCACCTCCCCAAATGCCTTTATGACTGCATCTCTTGAATCCTGGAGAG

GTGAGTGGAATTCCGAGTGTATAGGTGAAATTCGTAGATATTCTGAAGAACACCAGTGGCGAAAGCGGCTCACTGGACTGCTATTGACACTGAGGTGCGAAAACGTGTGGAGCAAACACGATTATATACCCTGATAGTCCCCGCCGCATACGATGATAACGAGCTGTCGGGGCTCTTATAGCTTCTGGTGGCGCACCTCACGCATTAATGTTATCCACCTGGGGAGTACGGCCGCCAGATTAAACTCAAATGAATTGACGGGGGCCTGCACAAGCGGTGGAGCATGTGGTTTAATTCCAACCAACGCGCAGAACTTACCAGCGTTTGACATGTCCGGTACGATTATCTGGAGACGATCTCTTTCCATCTGGGCACTGACACACAGTGCTGCATGTCTGTCGTCAACTCGTGTCGTGAGATGATGGGTTAGACCGCGACGACAGCACCCTCCTCCTTAAGTTGTCGTCAAT

**>Culture_D5_**

GCATGCAGCGAGCTATACATGCAAGTCGAACGAACGGAGGAAGAGCTTGCTCTTCCAAAGTTAGTGGCGGACGGGTGAGTAACACGTGGGCAACCTGCCTGTAAGTTGGGGATAACTCCGGGAAACCGGGGCTAATACCGAATGATAAAGTGTGGCGCATGCCACGCTTTTGAAAGATGGTTTCGGCTATCGCTTACAGATGGGCCCGCGGTGCATTAGCTAGTTGGTAGGGTAATGGCCTACCAAGGCAACGATGCATAGCCGACCTGAGAGGGTGATCGGCCACACTGGGACTGAGACACGGCCCAGACTCCTACGGGAGGCAGCAGTAGGGAATCTTCCGCAATGGACGAAAGTCTGACGGAGCAACGCCGCGTGTATGAAGAAGGTTTTCGGATCGTAAAGTACTGTTGTTAGAGAAGAACAAGGATAAGAGTAACTGCTTGTCCCTTGACGGTATCTAACCAGAAAGCCACGGCTAACTACGTGCCAGCAGCCGCGGTAATACGTAGGTGGCAAGCGTTGTCCGGATTTATTGGGCGTAAAGCGCGCGCAGGCGG

TCTTTTAAGTCTGATGTGAAAGCCCCCGGCTTAACCGGGGAGGGTCATTGGAAACTGGAAGACTGGAGTGCAGAAGAGGAGAGTGGAATTCCACGTGTAGCGGTGAAATGCGTAGATATGTGGAGGAACACCAGTGGCGAAGGCGACTCTCTGGTCTGTAACTGACGCTGAGGCGCGAAAGCGTGGGGAGCAAACAGGATTAGATACCCTGGTAGTCCACGCCGTAAACGATGAGTGCTAAGTGTTAGGGGTTTCCGCCCCTTAGTGCTGCAGCTAACGCATTAAGCACTCCGCCTGGGGAGTACGACCGCAAGGTTGAAACTCAAAGGAATTGACGGGGGCCCGCACAAGCGGTGGAGCATGTGGTTTAATTCGAAGCAACGCGAAGAACCTTACCAGGTCTTGACATCCTTTGACCACTCTGGAGACGGAGCTTCCCTTTCGGGGACAAGGTGACAGGTGGTGCATGGTTGTCGTCAG

CTCGTGTCGAT

**>Culture_H1_**

TGGATGGCGGCAGCTACACATGCAGTCGAGCGGATGAGAGGAGCTTGCTCCTGGATTCAGCGGCGGACGGGTGAGTAATGCCTAGGAATCTGCCTGGTAGTGGGGGACAACGTTTCGAAAGGAACGCTAATACCGCATACGTCCTACGGGAGAAAGCAGGGGACCTTCGGGCCTTGCGCTATCAGATGAGCCTAGGTCGGATTAGCTAGTTGGTGAGGTAATGGCTCACCAAGGCGACGATCCGTAACTGGTCTGAGAGGATGATCAGTCACACTGGAACTGAGACACGGTCCAGACTCCTACGGGAGGCAGCAGTGGGGAATATTGGACAATGGGCGAAAGCCTGATCCAGCCATGCCGCGTGTGTGAAGAAGGTCTTCGGATTGTAAAGCACTTTAAGTTGGGAGGAAGGGTTGTAGATTAATACTCTGCAATTTTGACGTTACCGACAGAATAAGCACCGGCTAACTCTGTGCCAGCAGCCGCGGTAATACAGAGGGTGCAAGCGTTAATCGGAATTACTGGGCGTAAAGCGCGCGTAGGTGGTTCGTTAAGTTGGA

TGTGAAATCCCCGGGCTCAACCTGGGAACTGCATTCAAAACTGTCGAGCTAGAGTATGGTAGAGGGTGGTGGAATTTCCTGTGTAGCGGTGAAATGCGTAGATATAGGAAGGAACACCAGTGGCGAAGGCGACCACCTGGACTGATACTGACACTGAGGTGCGAAAGCGTGTGGAGCAAACAGGATTAGATACCCTGGTAGTCCACGCCGTAAACGATGTCAACTAGCCGTTGGGAGCCTTGAGCTCTTAGTGGCGCAGCTAACGCATTAAGTTGACCGCCTGGGGAGTACGGCCGCAAGGTTAAAACTCAAATGAATTGACGGGGGCCCGCACAAGCGGTGGAGCATGTGGTTTATTTCGAAGCAACGCGAGAACTTACCAGGCCTTGACATCCAATGACTTTCAGAGATGGATTGTGCCTTCGGACATTGAGACAGGTGCTGCATGGCTGTCGTCAGCTCGTGTCGTGAGATGTTGGGTTAAGTCCCGTA

**>Culture_H3_**

GGTGCGGCAGCTACACATGCAGTCGAGCGGTAGAGAGAAGCTTGCTTCTCTTGAGAGCGGCGGACGGGTGAGTAATGCCTAGGAATCTGCCTGGTAGTGGGGGATAACGTTCGGAAACGGACGCTAATACCGCATACGTCCTACGGGAGAAAGCAGGGGACCTTCGGGCCTTGCGCTATCAGATGAGCCTAGGTCGGATTAGCTAGTTGGTGGGGTAATGGCTCACCAAGGCGACGATCCGTAACTGGTCTGAGAGGATGATCAGTCACACTGGAACTGAGACACGGTCCAGACTCCTACGGGAGGCAGCAGTGGGGAATATTGGACAATGGGCGAAAGCCTGATCCAGCCATGCCGCGTGTGTGAAGAAGGTCTTCGGATTGTAAAGCACTTTAAGTTGGGAGGAAGGGCAGTAAATTAATACTTTGCTGTTTTGACGTTACCGACAGAATAAGCACCGGCTAACTCTGTGCCAGCAGCCGCGGTAATACAGAGGGTGCAAGCGTTAATCGGAATTACTGGGCGTAAAGCGCGCGTAGGTGGTTCGTTAAGTTGGATGT

GAAATCCCCGGGCTCAACCTGGGAACTGCATTCAAAACTGACGAGCTAGAGTATGGTAGAGGGTGGTGGAATTTCCTGTGTAGCGGTGAAATGCGTAGATATAGGAAGGAACACCAGTGGCGAAGGCGACCACCTGGACTGATACTGACACTGAGGTGCGAAAGCGTGGGGAGCAAACAGGATTAGATACCCTGGTAGTCCACGCCGTAAACGATGTCAACTAGCCGTTGGGAGCCTTGAGCTCTTAGTGGCGCAGCTAACGCATTAAGTTGACCGCCTGGGAGTACGGCCGCAACGCTAAAACTCAAATGAATTGACGGGGGCCGCACAAGCGGTGGAGCATGTGTTTAATTCGAAGCAACGCGAAAACCTTACCAGGCCTTGACATCAATGAACTTTCTAGAGATAGATTGTGCCTTCGGTACATTGAGACAGGTGCTGCATGGCTGTCGTCAGCTCGTGTCGTGAGATGCTCGGTTAAGTTCCGTACGAACGCCAA

16S rRNA sequences obtained from Eurofins (Germany): 1492R (Reverse primer)

**>Culture_A3_**

CAAGTCTGATCAACCGTGGTACCGTCCTCCCGAAGGTTAGACTAGCTACTTCTGGTGCAACCCACTCCCATGGTGTGACGGGCGGTGTGTACAAGGCCCGGGAACGTATTCACCGTGACATTCTGATTCACGATTACTAGCGATTCCGACTTCACGCAGTCGAGTTGCAGACTGCGATCCGGACTACGATCGGTTTTATGGGATTAGCTCCACCTCGCGGCTTGGCAACCCTTTGTACCGACCATTGTAGCACGTGTGTAGCCCAGGCCGTAAGGGCCATGATGACTTGACGTCATCCCCACCTTCCTCCGGTTTGTCACCGGCAGTCTCCTTAGAGTGCCCACCATAACGTGCTGGTAACTAAGGACAAGGGTTGCGCTCGTTACGGGACTTAACCCAACATCTCACGACACGAGCTGACGACAGCCATGCAGCACCTGTCTCAATGTTCCCGAAGGCACCAATCCATCTCTGGAAAGTTCATTGGATGTCAAGGCCTGGTAAGGTTCTTCGCGTTGCTTCGAATTAAACCACATGCTCCACCGCTTGTGCGGGCCCCC

GTCAATTCATTTGAGTTTTAACCTTGCGGCCGTACTCCCCAGGCGGTCAACTTAATGCGTTAGCTGCGCCACTAAGAGCTCAAGGCTCCCAACGGCTAGTTGACATCGTTTACGGCGTGGACTACCAGGGTATCTAATCCTGTTTGCTCCCCACGCTTTCGCACCTCAGTGTCAGTATCAGTCCAGGTGGTCGCCTTCGCCACTGGTGTTCCTTCCTATATCTACGCATTTCACCGCTACACAGGAAATTCCACCACCCTCTACCATACTCTAGCTCGACAGTTTTGAATGCAGTTCCCAGTTGAGCCCGGGGATTTCACATCCAACTTAACGAACCACCTACGCGCGCTTTACGCCCAGTAATTCCGATTACGCTTGCACCCCTCTGTATTACCGCGGCTGCTGGCACAGAGTTAGCCGGTGCTTATTTCTGTCGGTAACGTCCA

**>Culture_D1_**

ATAAGATCGGTATACTATCGTGGTAAGCGTGCCTCCTTAACGTGTTAGACTGACACTTGACCTTCGGTTGCAACCCAACTCCCATGGTGTGACGGGCGGTGTGTACAAGGCCCGGGAACGTATTCACCGCGACTTGCTGATCGGATTACTAGCGATTCCACTTCATGATCTCAGTTGCAGACTGCAATCCGAACTGAGACCGCTTTTATGGAATTACCTCCCCCTCCCGGATTGCCGCCCCTTGGCACCGCCCTTTGAACCAGGGGGGAACCCCACGCCTAAAGGGCCAGGAGAACTTGACTCCTCCCCCCCCTTCCCCCGGTTTGTCACCGGCAGTCTCCTTAAAGTGCCCACCATAAGGTGCTGGTAACTGAGGAGGAGGGTTGCCCTCGTTGGAGTACTTACCCCAACATCTCACAACACCAGCTGACAACCGCGATGCACCAGCTGTCTCATCGTTCCCGAAAGCACAACTCTCTCTCTAGAAAGTTCGGTGGAGGTCAAGGCTGGGAAAGGTTCTTCCCGTTGCTTCAAATTAAACCCCAGGCCCCCCCCCTGGT

GCGGCCCCCCGCCAATTCTTTTGAGTTTTAACCTTGCGGCCGTATTCCCCAGGCGATCAACTTATGGCGTTACCTGCGCAACTAAGATCTCAAAGCCCCCAAAGGTAAGTTGACATCTTTTACGGGTGGAACTACCAGGGAATCTAATCCGGTTGCTCTCCCCACGCTTTCACACCTCACGGTCATTATCAGTCCAGGAGCCCGCCTCTCGCACACGGTGTGTTCCTTCAATATCTCTACACATTTCACTCGCTACTCGGAAATTCACACCACCTCTCTACGGTACTCAACGATGCCATCTTTAGAATGAAGTTTCCAGGTTAGACTCCGGGGGTTTTCCCTCTTAACTTTAAAAAAACCCCCTACATGCGCCCTTTAGCCCGTAATTTTCAAATAAACGTATCTCCCCCTCGTGATATACCCCCGGCTGTCTGGCCAGAGAAGTATATCGGAGGCTTAATCCTCGGCGGGAAAGGTCATTATCATTCCCCGTA

**>Culture_D5_**

CCGTAGCTGTCTACCATTCGGCGGCTGGCTCCATAAAGGTTACCCTACCGACTTCGGGTGTTACAAACTCTCGTGGTGTGACGGGCGGTGTGTACAAGGCCCGGGAACGTATTCACCGTGGCATGCTGATCCACGATTACTAGCGATTCCGGCTTCATGTAGGCGAGTTGCAGCCTACAATCCGAACTGAGAATAGTTTTATGGGATTAGCTCCACCTCGCGGCTTCGCGACCCTTTGTACTATCCATTGTAGCACGTGTGTAGCCCAGGTCATAAGGGGCATGATGATTTGACGTCATCCCCACCTTCCTCCGGCTTGCACCGGCAGTCACTTTAGAGTGCCCAACTAAATGCTGGCAACTAAAATCAAGGGTTGCGCTCGTTGCGGGACTTAACCCAACATCTCACGACACGAGCTGACGACAACCATGCACCACCTGTCACTTTGTCCCCGAAGGGAAAGCTCTGTCTCCAGAGTGGTCAAAGGATGTCAAGACCTGGTAAGGTTCTTCGCGTTGCTTCGAATTAAACCACATGCTCCACCGCTTGTGCGGGCCCCC

GTCAATTCCTTTGAGTTTCAACCTTGCGGTCGTACTCCCCAGGCGGAGTGCTTAATGCGTTAGCTGCAGCACTAAGGGGCGGAAACCCCCTAACACTTAGCACTCATCGTTTACGGCGTGGACTACCAGGGTATCTAATCCTGTTTGCTCCCCACGCTTTCGCGCCTCAGCGTCAGTTACAGACCAGAGAGTCGCCTTCGCCACTGGTGTTCCTCCACATATCTACGCATTTCACCGCTACACGTGGAATTCCACTCTCCTCTTCTGCACTCCAGTCTTCCAGTTTCCAATGACCCTCCCCGGTTAAGCCGGGGGCTTTCACATCAGACTTAAAAGACCGCCTGCGCGCGCTTTACGCCCAATAATCCGGACAACGCTTGCCACCTACGTATTACCGCGGCTGCTGGCACGTAGTTAGCCGTGGCTTTCTGGTTAGATACCGTCAGGGAACAAGCAGTTACCTCTAAT

**>Culture_H1_**

CAGAAGATAACCGTGGTACCGTCCTCCCGAAGGTTAGACTAGCTACTTCTGGTGCAACCCACTCCCATGGTGTGACGGGCGGTGTGTACAAGGCCCGGGAACGTATTCACCGTGACATTCTGATTCACGATTACTAGCGATTCCGACTTCACGCAGTCGAGTTGCAGACTGCGATCCGGACTACGATCGGTTTTATGGGATTAGCTCCACCTCGCGGCTTGGCAACCCTTTGTACCGACCATTGTAGCACGTGTGTAGCCCAGGCCGTAAGGGCCATGATGACTTGACGTCATCCCCACCTTCCTCCGGTTTGTCACCGGCAGTCTCCTTAGAGTGCCCACCATTACGTGCTGGTAACTAAGGACAAGGGTTGCGCTCGTTACGGGACTTAACCCAACATCTCACGACACGAGCTGACGACAGCCATGCAGCACCTGTCTCAATGTTCCCGAAGGCACCAATCCATCTCTGGAAAGTTCATTGGATGTCAAGGCCTGGTAAGGTTCTTCGCGTTGCTTCGAATTAAACCACATGCTCCACCGCTTGTGCGGGCCCCCGTC

AATTCATTTGAGTTTTAACCTTGCGGCCGTACTCCCCAGGCGGTCAACTTAATGCGTTAGCTGCGCCACTAAGAGCTCAAGGCTCCCAACGGCTAGTTGACATCGTTTACGGCGTGGACTACCAGGGTATCTAATCCTGTTTGCTCCCCACGCTTTCGCACCTCAGTGTCAGTATCAGTCCAGGTGGTCGCCTTCGCCACTGGTGTTCCTTCCTATATCTACGCATTTCACCGCTACACAGGAAATTCCACCACCCTCTACCATACTCTAGCTCGACAGTTTTGAATGCAGTTCCCAGTTGAGCCCGGGGATTTCACATCCAACTTAACGAACCACCTACGCGCGCTTTACGCCCAGTAATTCCGATTAACGCTTGCACCCTCTGTATTACCGCGGCTGCTGGCACAGAAGTTAGCCGGTGCTTATTCCTGTCGGTAACGTCAAAATTTGCAGAAGTTATTAATCTA

**>Culture_H3_**

CAGTGATCAACCGTGGTACCGTCCTCCGAAGGTTAGACTAGCTACTTCTGTGTGCAACCCACTCCCATGGTGTGACGGGCGGTGTGTACAAGGCCCGGGAACGTATTCACCGCGACATTCTGATTCGCGATTACTAGCGATTCCGACTTCACGCAGTCGAGTTGCAGACTGCGATCCGGACTACGATCGGTTTTCTGGGATTAGCTCCACCTCGCGGCTTGGCAACCCTCTGTACCGACCATTGTAGCACGTGTGTAGCCCAGGCCGTAAGGGCCATGATGACTTGACGTCATCCCCACCTTCCTCCGGTTTGTCACCGGCAGTCTCCTTAGAGTGCCCACCATTACGTGCTGGTAACTAAGGACAAGGGTTGCGCTCGTTACGGGACTTAACCCAACATCTCACGACACGAGCTGACGACAGCCATGCAGCACCTGTCTCAATGTTCCCGAAGGCACCAATCTATCTCTAGAAAGTTCATTGGATGTCAAGGCCTGGTAAGGTTCTTCGCGTTGCTTCGAATTAAACCACATGCTCCACCGCTTGTGCGGGCCCCCGTC

AATTCATTTGAGTTTTAACCTTGCGGCCGTACTCCCCAGGCGGTCAACTTAATGCGTTAGCTGCGCCACTAAGAGCTCAAGGCTCCCAACGGCTAGTTGACATCGTTTACGGCGTGGACTACCAGGGTATCTAATCCTGTTTGCTCCCCACGCTTTCGCACCTCAGTGTCAGTATCAGTCCAGGTGGTCGCCTTCGCCACTGGTGTTCCTTCCTATATCTACGCATTTCACCGCTACACAGGAAATTCCACCACCCTCTACCATACTCTAGCTCGTCAGTTTTGAATGCAGTTCCCAGTTGAGCCCGGGGATTTCACATCCAACTTAACGAACCACCTACGCGCGCTTTACGCCCAGTAATTCCGATTAACGCTTGCACCCCTCTGTATTACCGCGGCTGCTGGCACAGAGTTAGCCGGTGCTTATTCTTGTCGGTAACGTCAAACAGCAAAGTATTAATTTACTGCCC
